# Decomposing Brand Loyalty: An Examination of Loyalty Subcomponents, Product Price Range, Consumer Personality, and Willingness to Pay

**DOI:** 10.3390/bs15020189

**Published:** 2025-02-11

**Authors:** Gabriele Damaschi, Ali Aboueldahab, Marco D’Addario

**Affiliations:** Department of Psychology, University of Milano-Bicocca, 20126 Milan, Italy; g.damaschi@campus.unimib.it (G.D.); a.aboueldahab@campus.unimib.it (A.A.)

**Keywords:** brand loyalty, product price range, consumer personality, willingness to pay, attitudinal loyalty, behavioral loyalty

## Abstract

Brand loyalty is widely recognized as a key driver of consumer behavior, significantly influencing their willingness to pay more for preferred brands. This study explores the relationship between brand loyalty, its components, willingness to pay, consumer personality traits, and product price range. A tailored online survey involving participants aged 18 to 60 revealed a strong positive correlation between brand loyalty and increased willingness to pay. The findings also underscore the influence of Conscientiousness and Energy on brand loyalty, alongside the moderating effect of product price range. Specifically, habit-based loyalty dominates low-priced, routine purchases, while higher-priced decisions involve deeper cognitive-affective evaluations. These insights offer valuable guidance for enhancing customer satisfaction and optimizing brand profitability.

## 1. Introduction

Brand loyalty plays a fundamental role in understanding consumer behavior. It refers to the relationship stakeholders develop with a brand, characterized by repeated purchases, active engagement, brand advocacy, and co-creation of value (Parris & Guzmán, 2023). As a key construct in consumer behavior research, brand loyalty is also a central component of brand equity (Aaker, 1991), which reflects a brand’s capacity to influence consumer decisions. This influence stems from both tangible attributes, such as product quality, and intangible factors, including psychological associations and emotional connections.

Despite the extensive literature on brand loyalty, significant gaps remain in understanding the underlying psychological mechanisms that drive these relationships, particularly in the context of how they interact with individual consumer differences and product-specific variables (Oliver, 1999; Dick & Basu, 1994).

Considering brand loyalty as a relationship between brand and consumer allows one to focus on the mutual benefits derived for both parties. This perspective has been increasingly recognized as essential for advancing theoretical frameworks that integrate both consumer psychology and market dynamics (Chaudhuri & Holbrook, 2001). A significant aspect of brand loyalty’s importance lies in its predictive power for behavioral outcomes such as willingness to pay, which can be defined as the maximum price a consumer is willing to pay for a certain number of goods or services (Katt & Meixner, 2020). Research has long established that higher brand loyalty leads to greater tolerance for product pricing, as demonstrated by Kalyanaram and Little (1994) and later confirmed by Kabadayi and Aygün (2007). Numerous studies, including Santos and Schlesinger (2021) and Malarvizhi et al. (2022), have demonstrated that brand loyalty drives the consumer to pay a premium for their favorite brands. In terms of the brand-consumer relationship, psychological constructs such as Brand Trust (e.g., Atulkar, 2020), Brand Affect (e.g., Chaudhuri & Holbrook, 2001), Brand Attachment (e.g., Özer et al., 2022), and Brand Love (e.g., Le, 2021) have been conceptualized in order to explore the personal significance that brands may hold for consumers’ self-expression (e.g., Munteanu & Andreea, 2014; Chieng et al., 2022; Boateng et al., 2020; Sarkar et al., 2021; Loh et al., 2021). While several studies have identified some subcomponents of brand loyalty, there is no unanimous agreement on its exact structure. Moreover, there is an insufficient amount of research systematically examining how these constructs interconnect within broader frameworks of consumer decision-making, especially in relation to individual psychological traits and contextual factors.

Aligned with this type of study, there is a line of research aimed at further investigating the psychological mechanisms facilitating the consumer-brand relationship. This involves examining how individual differences, such as psychological traits and behavioral tendencies, influence consumer loyalty (Mittal & Kamakura, 2001). Additionally, it is important to consider the role of contextual factors, such as product type and price range (e.g., mid-to-high-end versus low-end products), and how these variables interact with individual differences to shape consumer decision-making and loyalty behaviors. While prior studies have offered valuable insights, they often lack a comprehensive integration of these factors (Zeithaml, 1988), leaving critical questions unanswered regarding their interplay. Despite growing interest in these topics, the interplay between consumer personality, contextual factors such as product price range, and brand loyalty remains underexplored and requires further investigation. This study aims to address these gaps by offering a nuanced perspective on the multifaceted nature of brand loyalty, with the potential to inform both theoretical development and practical applications.

### 1.1. Internal Structure of Brand Loyalty

Dick and Basu (1994) have conceptualized customer loyalty—a broader concept than brand loyalty, but with shared subcomponents (Ishak & Abd Ghani, 2013; Singh, 2021)—as consisting of two key dimensions: an attitudinal component, referred to as Relative Attitude and a behavioral component, referred to as Repeat Patronage. Similarly, Knox and Walker (2001) identified two comparable dimensions, which they termed “Commitment” and “Support”, while Delgado-Ballester and Munuera-Alemán (2001) contributed to this framework by introducing the corresponding concepts of “Customer Commitment” and “Brand Repurchase Intention”. These consistent findings led to the development of a model of brand loyalty defined by two distinct dimensions: attitudinal loyalty and behavioral loyalty, also referred to as “Purchase Loyalty” or “Action Loyalty.” Attitudinal loyalty reflects the emotional and cognitive commitment a consumer has toward a brand, including their preferences and intentions. Behavioral loyalty, on the other hand, refers to the tangible actions taken by consumers, such as repeat purchases and ongoing engagement. Together, these dimensions form the foundation of brand loyalty, integrating both psychological attachment and observable behavior. This dual-structure model has been validated by numerous studies (e.g., Chaudhuri & Holbrook, 2001; Evanschitzky et al., 2006; Gecti & Zengin, 2013; Y. G. Choi et al., 2017; Jiddi, 2023). Moreover, several studies have found that attitudinal loyalty predicts behavioral loyalty, but not vice versa (e.g., Saini & Singh, 2020; Dick & Basu, 1994; Evanschitzky et al., 2006; Gecti & Zengin, 2013; Y. G. Choi et al., 2017). In other words, a positive attitude toward a specific brand, rooted in emotional and cognitive commitment, often translates into loyal behaviors such as repeated purchases and word-of-mouth promotion.

However, this dual-structure model has faced criticism for its oversimplification of the complex, multidimensional nature of brand loyalty. Some recent models have introduced new layers of complexity. For example, the Brand Attachment Theory focuses on the strong bonds consumers develop with brands, which can include emotional, symbolic, or functional connections. It suggests that stronger attachments lead to greater loyalty (e.g., Park et al., 2010). This theory deepens our understanding of loyalty by emphasizing the emotional connections between consumers and brands, which may not be fully captured in the attitudinal and behavioral loyalty framework. Nevertheless, it (Brand Attachment Theory) may overlook situational or contextual factors that can influence loyalty (e.g., changes in brand positioning or market conditions).

Some studies have identified more than two dimensions within brand loyalty, although finding less consensus among authors. For example, Worthington et al. (2010) proposed a model of brand loyalty consisting of three dimensions: behavioral, affective, and cognitive. Another widely cited study by Oliver (1999) described four progressive phases of loyalty: cognitive, affective, conative, and action loyalty. This model has also been revisited in more recent works (e.g., El-Manstrly & Harrison, 2013; X. Han et al., 2008; H. Han et al., 2011; Taoana et al., 2021; Javed et al., 2023). Oliver’s model assumes a linear progression through the stages, which may not always apply in today’s fast-paced digital environment in which emotional, cognitive, and behavioral factors interact in complex and overlapping ways. Additionally, the influence of social media and algorithm-driven personalized digital content has added new complexities to understanding brand loyalty that traditional models may not fully address (e.g., Kumar et al., 2022).

Dick and Basu (1994) introduced related dimensions within brand loyalty, distinguishing between Relative Attitude, which reflects consumers’ overall evaluation of a brand, and Repeat Patronage, which refers to their consistent purchasing behavior. They emphasized the role of cognitive (thoughts), affective (feelings), and behavioral intentions in shaping consumer loyalty, aligning with Eagly and Chaiken’s (1998) tripartite model of attitudes. This framework, while robust in integrating psychological antecedents of loyalty, overlooks the emerging role of digital platforms. Recent research highlights how social media’s personalized brand interactions and advertising models influence consumer loyalty (e.g., Huang & Benyoucef, 2013). The Social Media Influence Model, for instance, demonstrates that both brand-driven content and user-driven interactions on social media can foster relationships with consumers, build trust, and cultivate long-term brand loyalty, particularly among younger, digitally engaged audiences.

An interesting model was proposed by Nordhielm and Williams (2008), which was later used by Dapena-Baron et al. (2020) to develop a specific measurement scale. These authors identified three components of brand loyalty: an affective component (Heart Loyalty), a cognitive component (Head Loyalty), and a habit-based component (Hand Loyalty). The first one corresponds to an intense emotional connection with a certain brand, the second to a favorable evaluation of the quality and features of the brand’s products or services, while the third is associated with the mere habit of purchasing from the same brand without any emotional or cognitive involvement. According to this model, the same consumer may exhibit different levels of Heart, Head, and Hand Loyalty across different brands, and a single brand may have consumers whose loyalty is driven more by affective, cognitive, or habitual factors. This model expands our understanding of brand loyalty by introducing a scale and identifying three specific factors that address aspects previously overlooked. In this framework, the brand connection can be more conative-behavioral, meaning that loyalty may sometimes be driven by habitual actions rather than emotional or cognitive engagement. Over time, purchasing from a certain brand can become habitual, reducing cognitive involvement; for instance, consumers may stop checking product details. This may also occur on an emotional level, leading to a decrease in emotional involvement toward the brand over time. Some brands, lastly, are considered more ordinary than others, and their products, once incorporated into daily life, may lose their salience (Coupland, 2005). Despite this, consumers with high habit-based loyalty continue to purchase these products regularly and remain satisfied with their choice. According to Dapena-Baron et al. (2020), this is not simply inertia in purchase decisions but represents a real form of loyalty driven by habit, as the long-term relationship established with the brand remains characterized by a clear preference for it.

In conclusion, the dual-structure model of brand loyalty, which includes attitudinal loyalty (ABL) and behavioral loyalty (BBL), has proven to be a robust and comprehensive framework for examining brand loyalty, making it the most suitable for this study. The model’s focus on the psychological and behavioral dimensions of loyalty offers a clear and actionable understanding of consumer behavior. While more recent models, such as those incorporating social media influence or emotional attachment, have expanded our understanding of loyalty in the digital era, the ABL and BBL frameworks provide a strong foundation for examining brand loyalty in traditional and contemporary contexts. Future studies may consider incorporating elements from newer models to explore the evolving dynamics of consumer-brand relationships in more digitally integrated environments.

### 1.2. Brand Loyalty, Product Price Range, and Personality

There is significant and consistent evidence that personality traits strongly influence individual consumer behaviors, with studies showing a clear correlation between specific traits and consumption patterns (Liu et al., 2017). However, the relationship between consumer personality and brand loyalty remains unclear, as authors studying this topic have not reached a unanimous conclusion. Several authors have utilized the Big Five model (McCrae & Costa, 1985, 1987, 2003, 2013; Costa & McCrae, 1999), which considers personality as being comprised of five traits: Neuroticism, openness to experience, Agreeableness, Conscientiousness, and Extraversion (it is worth noting that some scholars employ different terminology to refer to these same traits). Matzler et al. (2006) found that the traits of openness to experience and Extraversion are strong predictors of Brand Affect, which in turn influences both attitudinal and behavioral outcomes, particularly attitudinal loyalty and purchase loyalty. Lin (2010) considered Brand Personality—the construct describing a brand’s character—as a moderating variable in the relationship between consumer personality and brand loyalty, distinguishing between the preference one feels toward a certain brand (affective loyalty) and the related behaviors (Action Loyalty); the results indicated that both openness to experience and Agreeableness influence affective and Action Loyalty. Durukan and Bozaci (2011) identified openness to experience, Agreeableness, and Emotional Stability as traits that positively correlate with customer loyalty. Leon and Dixon (2023) state that openness to experience and Agreeableness positively influence customer satisfaction, which in turn could lead to loyalty. Ho and Wong (2023) demonstrated that more conscientious, extroverted, and agreeable consumers tend to have greater trust in a particular banking service, which in turn results in higher attitudinal and behavioral loyalty. Azzahra et al. (2024) argue that the traits impacting customer loyalty are openness to experience, Extraversion, and Emotional Stability, the latter being associated with calmness and resilience.

Overall, the studies discussed above suggest that dividing brand loyalty into attitudinal and behavioral components does not sufficiently explain how individual consumer personality influences brand loyalty or identify which personality traits are most relevant. Researchers have yet to reach a consensus, as different studies highlight varying combinations of traits. This lack of clarity can also be attributed to the limited number of studies on this topic. Given that the relationship between brand loyalty and personality traits has not yet been extensively studied, drawing definitive conclusions about which traits predispose individuals to form deeper connections with brands is challenging and remains an area for further research.

The work of Bove and Mitzifiris (2007) diverges from the other mentioned studies because, instead of identifying which individual traits are more or less associated with brand loyalty, they conclude that consumer personality does not necessarily influence the formation of a deep bond with brands. These authors proposed that the lack of significant findings concerning the relationship between personality and brand loyalty could be partially attributed to low-involvement research contexts, as they hypothesized in their study on loyalty to fast-food chains. According to Odekerken-Schröder et al. (2003), a relationship between personality and brand loyalty can only be established when the product and the category it belongs to generate a high enough level of involvement. In other words, personality traits are more likely to influence brand loyalty when the individual is deeply engaged with the product or brand, as in situations where the product is important to them or requires careful consideration. So, in high-involvement contexts, such as during the decision-making process for the purchase of expensive goods, the influence of individual differences might be more pronounced and measurable. On the other hand, in a low-involvement context, such as everyday or low-cost purchases, this relationship may be weaker or less evident. However, there are still a few studies that address this topic, particularly in relation to product price range, which could influence the nature or intensity of the bond between the individual and their preferred brands by affecting individual involvement.

### 1.3. Research Objectives

The primary objective of this study is to examine the nature of the relationship between brand loyalty (BL), consumer personality, and willingness to pay (WTP) across two distinct price categories: low and mid-high. Consumer personality is conceptualized using the Big Five model. Brand loyalty is analyzed by dividing it into behavioral loyalty (BBL) and attitudinal loyalty (ABL), which further encompasses affective loyalty (aABL), cognitive loyalty (cABL), and habit loyalty (hABL).

Regarding the hypotheses formulated a priori, the initial goal is to assess the relationship between ABL and BBL, consistent with the literature.

**Hypothesis** **1:** *Attitudinal Loyalty regresses onto Behavioral Loyalty*.

Another aim of this study is to clarify the relationship between the consumer personality traits, described in terms of the Big Five, and the components of brand loyalty, specifically BBL and ABL, including its subcomponents aABL, cABL, and hABL. Specifically, it is expected that various personality traits will influence the individual components of brand loyalty in different ways, as they may predispose consumers toward the more affective, cognitive, habitual, or behavioral aspects of the relationship with brands. Given the limited literature on this topic, no specific hypotheses are advanced regarding the associations between different personality traits and individual brand loyalty components.

**Hypothesis** **2:** *Personality traits are differentially associated with the individual components of Brand Loyalty.*

Furthermore, this study aims to test the tendency of loyal consumers to spend more on products and services offered by their preferred brands, in line with the literature. The concept of willingness to pay (WTP) has been adapted to “Willingness to Pay More” (WTPM) to emphasize the intention of brand-loyal consumers to spend a greater amount of money to obtain a product from their favorite brand rather than a similar one offered by competitor brands.

**Hypothesis** **3:** *Brand Loyalty regresses onto Willingness to Pay More.*

Previous research has not clarified whether the different components of brand loyalty are associated differently with WTPM. Therefore, another objective of this study is to highlight this point. The expectation is that the individual components of brand loyalty influence WTPM in different ways, depending on the type of relationship established between the consumers and their favorite brand. Due to the scarcity of evidence in the literature, no specific hypotheses are proposed regarding the associations between the individual components of brand loyalty and WTPM.

**Hypothesis** **4:** *The individual components of Brand Loyalty are differently associated with Willingness to Pay More.*

Lastly, the study aims to assess the influence of product price range on both brand loyalty and consumers’ WTPM. Specifically, it is expected that higher product prices will correspond to generally higher BL (ABL, BBL, aABL, cABL, but not hABL) and WTPM scores. It is hypothesized that hABL will show higher scores for the low-price range since, by definition, it concerns purchases where there are no significant emotional or cognitive factors, aspects that might be less present when the product cost is low.

**Hypothesis** **5:** *Brand Loyalty toward brands of mid-high price range products is significantly higher than that for low-price range brands across all its components (Attitudinal, Behavioral, Affective, and Cognitive Loyalty), except for Habit Loyalty, which is expected to have higher scores for the low-price range.*

**Hypothesis** **6:** *The Willingness to Pay More for mid-high price range brands is significantly higher than that for low-price range brands.*

**Figure 1:**
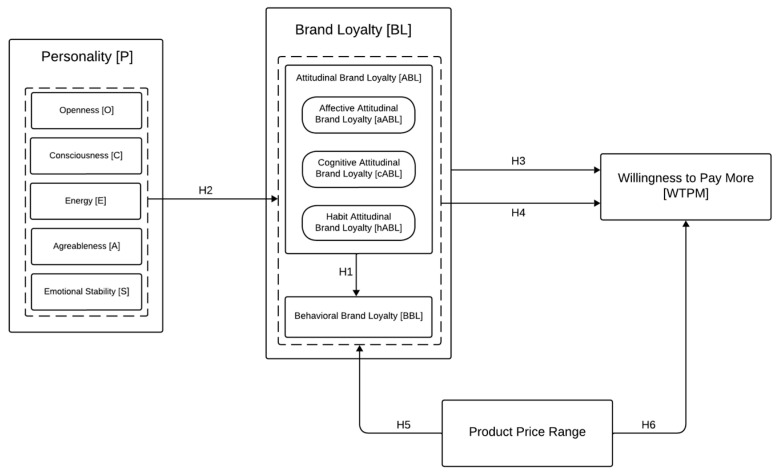
The Conceptual Model.

The conceptual framework of the study, along with the six hypotheses, is depicted in Figure 1.

## 2. Materials and Methods

### 2.1. Participants

The sample consisted of 347 participants aged between 18 and 60, recruited via social media (Instagram, LinkedIn) and messaging apps (WhatsApp). In terms of gender distribution, 62.5% of the participants identified as female, 36.6% as male, 0.6% selected “Other”, and 0.3% preferred not to specify. Participants were categorized into selectable age groups: 18–25, 26–30, 31–35, 36–40, and over 40. Of the participants, 56.2% were aged between 18 and 25, while the remaining 43.7% were aged between 26 and 60. Additionally, participants indicated their employment status by choosing from the following options: Student, Working Student, Employed, and Unemployed; the sample consisted of 63.1% workers and 36.9% non-workers.

The sole exclusion criterion was the absence of any preferred brands for the proposed products in either of the two price ranges.

### 2.2. Online Survey

The study employed a quantitative approach utilizing a custom-designed online survey administered through Qualtrics. Completion took between 15 and 30 min. The instrument comprised a first section relating to demographic information on gender, age, and employment status. Participants were then asked to choose, from a selection of three products, the one for which they had the most relevant favorite brand—or, in any case, the one they purchased from most often—and to write the name of the brand. These products were low-priced: toothpaste, non-alcoholic beverages, and cookies. The choice of these products was informed by their frequent use in prior studies on brand loyalty and consumer behavior (e.g., Chaudhuri & Holbrook, 2001; Dapena-Baron et al., 2020). These items represent everyday necessities that are typically low-involvement purchases, making them suitable for evaluating habitual and emotional loyalty patterns. Based on the brand linked to the selected product, participants responded to 6 items measuring aABL, 5 measuring cABL, and 5 measuring hABL. All 16 of these items were taken from the pre-existing, validated questionnaire by Dapena-Baron et al. (2020). Another 5 items were used to measure BBL and were drawn from another previously validated questionnaire used in Y. G. Choi et al. (2017). Participants were then shown two ad hoc items to measure WTPM. The first item (WTPMi) asked participants if they were willing to pay more to obtain the product offered by their favorite brand instead of a similar product from competitor brands. If they answered affirmatively, the second item (WTPMq) asked them to quantify, in percentage, the amount they were willing to pay more using a slider. At this point, a new selection of products was proposed, this time from the mid-high price range: cell phones, shoes, and luggage (bags, backpacks, suitcases). These products were selected due to their relevance in assessing both cognitive and emotional components of brand loyalty in higher-involvement purchasing decisions, as highlighted in studies by Matzler et al. (2006) and Chaudhuri and Holbrook (2001). Their broader price range and variability in purchase frequency make them suitable for capturing differences in consumer loyalty across product categories. The sections measuring brand loyalty components and WTPM were then repeated as described above. For the personality assessment, a brief version of the Big Five Adjectives (Barbaranelli et al., 2007) was used.

To ensure the reliability and validity of the instruments, a pre-testing phase was conducted prior to administering the survey. The pre-test involved 20 participants who were representative of the target population. Based on their feedback, minor adjustments were made to ensure clarity and usability. Furthermore, the internal consistency of the scales used in the study was verified using Cronbach’s alpha, with values exceeding the commonly accepted threshold of 0.70 for all constructs (Nunnally & Bernstein, 1994). This step ensures that the instruments reliably measure the intended constructs.

The decision to include the selected products was also influenced by their widespread use in prior research on brand loyalty and willingness to pay (e.g., Dapena-Baron et al., 2020; Matzler et al., 2006). These products were chosen not only for their frequent purchase and general familiarity among consumers but also for their ability to capture diverse aspects of loyalty across different pricing segments. This ensures the robustness of the findings and enhances their generalizability. The measurement items for BL subcomponents, WTPM and personality, can be found in Appendix A.

### 2.3. Data Analyses

Data analysis was conducted using the SPSS statistical package, vers. 29 (SPSS Inc., Chicago, IL, USA). Data is cointained within Supplementary Materials. The analyses performed included correlations, simple and logistic linear regressions, paired and independent samples *t*-tests, and ANOVAs (Analysis of Variance). For inferential analyses, a significance level of *p* < 0.05 was set a priori (Gallucci et al., 2017).

The analyses also resulted in the creation of several variables. aABL, cABL, hABL, and BBL were obtained by calculating the mean of the responses to the corresponding items, according to the item organization in Dapena-Baron et al. (2020) and Y. G. Choi et al. (2017). ABL was calculated by calculating the mean of aABL, cABL, and hABL, while overall BL corresponded to the mean of ABL and BBL. aABL, cABL, hABL, ABL, BBL, and BL were calculated in this way for both the low and mid-high price ranges. The mean of these variables for the two product price ranges was then calculated to obtain a general measure of these constructs. Personality trait scores were also calculated by calculating the mean of the responses to the single items, organized within the traits of Openness (O), Conscientiousness (C), Energy (E), Agreeableness (A), and Emotional Stability (S), as in Barbaranelli et al. (2007). Finally, the variables of aABL, cABL, hABL, ABL, BBL, BL, and WTPM, for both the low and mid-high price ranges and in general, as well as the Big Five, were standardized to enable comparisons between them.

## 3. Results

First, the relationship between ABL and BBL was investigated using a simple linear regression model, with ABL as the dependent variable and BBL as the independent variable. As shown in Table 1, H1 was confirmed, as ABL significantly regresses onto BBL for both product price ranges and overall.

A multiple linear regression model was then employed, keeping BBL as the dependent variable and replacing ABL with aABL, cABL, and hABL as the new independent variables, with the aim of verifying whether the relationship would hold when ABL is broken down into its affective, cognitive, and habit-based subcomponents. As can be seen in Table 2, significant associations between ABL subcomponents and BBL have been found.

H2 hypothesized that consumer personality traits would be differentially associated with BL components, without predicting specific trait-component associations due to the limited literature on this subject. Several correlations have been conducted, and their results suggest that H2 is only partially confirmed, as a clear differentiation in the influence of only two traits—C and E—was observed for almost all BL components. The significant correlational analyses demonstrated that:

- for low-priced products, more conscientious individuals show higher scores for cABL (*r* = 0.214 ***^1^), BBL (*r* = 0.199 ***), and overall BL (*r* = 0.148 *);
- for mid-high priced products, more conscientious individuals show higher scores for aABL (*r* = 0.135 *), cABL (*r* = 0.134 *), ABL (*r* = 0.143 *), BBL (*r* = 0.159 **), and overall BL (*r* = 0.163 **); more energetic individuals show higher scores for aABL (*r* = 0.184 ***), ABL (*r* = 0.149 **), BBL (*r* = 0.184 ***), and overall BL (*r* = 0.176 **), and more agreeable individuals show higher scores for cABL (*r* = 0.117 *);
- in general, more conscientious individuals show higher scores for aABL (*r* = 0.131 *), cABL (*r* = 0.218 ***), ABL (*r* = 0.154 *), BBL (*r* = 0.235 ***), and overall BL (*r* = 0.193 **); while more energetic individuals show higher scores for aABL (*r* = 0.136 *), ABL (*r* = 0.132 *), BBL (*r* = 0.177 **), and overall BL (*r* = 0.158 **).

Concerning H3, a logistic regression model was used to examine the causal relationship between BL and WTPM, where BL was entered as the independent variable and WTPMi (in which participants could indicate whether or not they were willing to pay a higher amount for the product offered from their favorite brand) was the dependent variable. As shown in Table 3, H3 was fully confirmed, as BL significantly predicts WTPMi for both low and mid-high price ranges. Consumers with stronger brand loyalty are more willing to pay higher prices for their preferred brands.

Logistic regression was also employed to verify whether the various components of BL influence the intention to pay more for one’s favorite brands, according to H4. A series of logistic regression models were tested, with WTPMi as the dependent variable and each BL subcomponent as the independent variable, which were analyzed individually. As shown in Table 4, for both low and mid-high price ranges, higher scores in aABL, cABL, ABL, and BBL are associated with greater consumer willingness to pay for their preferred brands. Conversely, higher hABL scores result in a lower WTPMi for the low price range, while the influence of hABL on WTPMi in the mid-high price range is not significant.

Next, the relationship between BL components and WTPMq was examined. The participants who answered affirmatively to WTPMi were then asked by WTPMq to quantify the percentage of the price premium they were willing to pay. A series of simple linear regression models were used to test whether the individual components of BL, as well as BL itself, influence the amount of the price premium loyal consumers are willing to spend on their favorite brand. Based on the analyses identified as significant, BBL predicts WTPMq for the low-price range (β = 0.175 *), while cABL predicts WTPMq for the mid-high price range (β = 0.158 *) and overall (β = 0.163 *).

Using paired *t*-tests, the significance of the difference in the statistical means of BL and its components between the low and mid-high price ranges was tested among participants who responded to the questionnaire sections for both product price ranges. H5 was fully confirmed, and the results clearly indicate that the price range of a brand’s products influences the level of BL. Specifically, mid-high-priced brands generate higher levels of aABL (*t* = −13.326 ***), cABL (*t* = −7.990 ***), ABL (*t* = −9.142 ***), BBL (*t* = −5.223 ***), and overall BL (*t* = −7.808 ***), and lower levels of hABL (*t* = 4.384 ***) compared to low-priced brands.

Concerning the relationship between product price range and WTPM, 66.78% of the participants answered affirmatively to WTPMi in the condition of low price range, while 75.16% of the participants did so for the mid-high price range; those willing to pay more for low-priced products would pay, on average, 25.7% more (M = 25.690, SD = 18.715), while those willing to pay more for mid-high-priced products would pay, on average, 32% more (M = 32.030, SD = 20.787).

Two paired *t*-tests were conducted to test the significance of the difference in means for the two WTPM items across the two product price ranges among participants who responded to both product price range sections. H6 was confirmed, as both the intention to pay a premium (*t* = −3.009 **) and its amount (*t* = −2.890 **) were significantly different for the two product price ranges, being higher for mid-high priced products.

Two exploratory analyses have been conducted in order to examine topics with a limited literature.

An exploratory analysis investigated the relationship between socio-demographic data, such as age and employment status, with BL and its component scores, as well as with WTPM scores. For this purpose, several within-subject univariate ANOVAs were conducted. Overall, participants under 25 years old showed significantly higher hABL scores compared to those over 25, across both low (*F* = 9.066 **) and mid-high (*F* = 11.172 ***) price ranges, as well as overall (*F* = 17.893 ***). Similarly, non-workers showed significantly higher hABL scores compared to workers across both low (*F* = 7.815 **) and mid-high (*F* = 5.782 *) price ranges, as well as overall (*F* = 10.141 **).

The second exploratory analysis focused on the relationship between personality and WTPM, a topic with limited literature. Correlation analyses were conducted for both WTPMi and WTPMq. The results show that more conscientious individuals would be more willing to pay more for low-priced products offered by their favorite brands (*r* = 0.162 **) but would pay a smaller premium for mid-high-priced products (*r* = −138 *). Conversely, more open individuals would pay a higher premium for products in this price range (*r* = 0.130 *).

## 4. Discussion

### Interpretation of the Results

The study’s general objective of testing the relationship between brand loyalty, product price range, consumer personality, and willingness to pay was partially achieved. Some personality traits were found to significantly influence brand loyalty and its components, which in turn significantly predicted the willingness to pay more. Moreover, product price range appeared to have a significant impact on the levels of both brand loyalty and willingness to pay more. The key findings are summarized, interpreted, and discussed below in relation to the initial hypotheses, previous studies, and key works in literature.

H1 has been confirmed, indicating that attitudinal loyalty is strongly associated with behavioral loyalty; specifically, higher levels of attitudinal loyalty are linked to higher levels of behavioral loyalty. Thus, a highly favorable attitude toward a particular brand drives consumers to exhibit their loyalty by engaging in behaviors such as repeat purchases and word-of-mouth. The confirmation of H1 aligns with the relevant literature (e.g., Dick & Basu, 1994; Evanschitzky et al., 2006; Y. G. Choi et al., 2017; Gecti & Zengin, 2013; Saini & Singh, 2020). This result is also consistent with two psychological classics, namely, the Theory of Reasoned Action (TRA; Fishbein & Ajzen, 1975; Ajzen, 1980) and the Theory of Planned Behavior (TPB; Ajzen, 1985, 1991, 2002), which assert that behavior enactment is influenced by the individual’s attitude toward that behavior.

The relationship between attitudinal and behavioral loyalty has also been investigated by distinguishing the subcomponents of attitudinal loyalty (affective, cognitive and habit). Both affective and cognitive loyalty significantly influence behavioral loyalty across product price ranges (low vs. mid-high), while habit loyalty does not. This suggests that customers who develop a positive emotional attachment (affective) or evaluate a brand’s technical attributes favorably (cognitive) are more likely to undertake loyalty behaviors. In contrast, habit loyalty alone is insufficient to guarantee such behaviors, as habitual purchasing may indicate current satisfaction but not necessarily future loyalty. For example, a consumer might decide to switch brands after discovering one with a better offer or one that is more emotionally appealing. This aligns with Aaker’s (1991) description of habitual buyers, who keep purchasing from a brand not because they are satisfied or engaged, but simply because they are not dissatisfied. However, these consumers may switch brands if a competitor’s offer is attractive enough to outweigh the perceived switching costs.

H2 has been only partially confirmed, as the only personality traits from the Big Five model that showed associations with brand loyalty and its components were Conscientiousness, Energy and Agreeableness. It is possible to infer that certain characteristics within these traits explain the observed relationships. For highly conscientious individuals, loyalty to their favorite brands, meant both as a favorable attitude toward a certain brand and as loyalty behaviors, may be based on their particular traits such as reliability, discipline, meticulousness, and organizational skills. For energetic individuals, brand loyalty may be driven by sociability and emotional attachment. Highly agreeable individuals, known for being cooperative and loyal, may naturally develop stronger loyalty ties; however, it remains unclear why these traits were specifically linked only to the cognitive component of brand loyalty. The most notable finding is that habit loyalty did not show any association with any of the personality traits, suggesting that this type of loyalty operates independently of individual personality. If this could be confirmed by future research, it would be an important insight for brand loyalty literature.

H3 has been confirmed, as brand loyalty overall predicts willingness to pay more, consistently with the literature (e.g., Santos & Schlesinger, 2021; Malarvizhi et al., 2022). This suggests that the stronger the connection a consumer feels with a brand, the more likely they are to be willing to pay a premium for its products.

H4 has been confirmed too, as brand loyalty components influence willingness to pay more (WTPMi, the intention to pay more to obtain the product offered by their favorite brand instead of a similar product from competitor brands). Specifically, consumers who demonstrate a highly favorable attitude—both cognitive and affective—toward a particular brand are more likely to pay a premium for that brand’s products, whether these are low or mid-high priced. This means that the more positively a person perceives the characteristics and qualities of a product, or the more emotionally attached they are to it, the more they will be inclined to pay more. In contrast, a strong habit of purchasing from a low-priced brand leads consumers to show a lower intention to pay more for that brand. Meanwhile, habitual purchasing from a brand that offers mid- to high-priced products does not significantly affect this willingness.

Moreover, it seems that brand loyalty components also influence the amount of money consumers are willing to pay more (WTPMq, the quantification of the premium expressed as a percentage of the additional amount a consumer is willing to pay to obtain the product offered by their favorite brand instead of a similar product from competitor brands). Overall, whether for low-priced or mid-high-priced products, the cognitive component of brand loyalty seems to have the greatest impact on this willingness to pay a premium as a behavioral outcome. Specifically, the more consumers believe a product has superior characteristics and qualities, the more likely they are to pay a higher price. Meanwhile, different psychological mechanisms appear to be activated for low-priced products when examining the different product price ranges. Here, the behavioral component of brand loyalty plays a significant role, since consumers who exhibit favorable behaviors toward a brand, such as consistently choosing it as their first option and encouraging others to purchase it, tend to pay a higher premium. In contrast, for mid-high-priced brands, the cognitive component remains predominant. This means that the more consumers evaluate mid-high-priced products positively in relation to their objective qualities, the more willing they are to pay a higher premium for it. This suggests that the cognitive aspects are primarily activated when determining how much more a consumer is willing to pay for their preferred brand; while this factor is less critical for low priced products, it becomes more significant with mid-high-priced items.

H5 was confirmed, showing a clear variation across brand loyalty (including its subcomponents) based on price range. Participants demonstrated higher brand loyalty scores for mid- to high-priced brands compared to low-priced ones, likely due to greater involvement and stronger connections with higher-priced brands, as suggested in the literature (e.g., Ferreira & Coelho, 2015; Quester & Lin Lim, 2003). Another confirmation of H5 comes from the habit subcomponent. In this case, consumers tend to have higher levels of habit loyalty for lower-priced products, which aligns with previously collected data. For mid- to high-priced products, cognitive and affective components play a more prominent role in shaping loyalty. In summary, brand loyalty levels are higher for mid- to high-priced products, with the exception of habit, which is more prevalent for low-priced items. This makes sense, as it is possible that lower-priced products involve less risk, making habitual purchases more common. In contrast, higher-priced products require more thought, leading to higher involvement and stronger cognitive and emotional loyalty. Thus, products from different price ranges could elicit different evaluations during the decision-making process related to purchasing.

H6 has been confirmed, since willingness to pay more levels significantly differ between low and mid-high price ranges. Specifically, customers are more inclined to pay extra and spend a larger premium for mid- to high-priced products compared to those that are low-priced. It may be that purchasing expensive products engages customers more, encouraging them to invest even more resources in order to obtain those goods. For this reason, loyal consumers may be willing to pay even more for those already high-priced products in order to remain loyal to their favorite brands.

Other relevant findings that deserve to be discussed are the following. Concerning the influence of age and employment status on brand loyalty and its subcomponents, it was observed that only the habit-based component showed significant relationships with age and employment status, which could be seen as indirect indicators of economic availability in the absence of specific income measures. This suggests that habit loyalty, may be more significant for consumers with lower financial means, such as younger individuals or those who are unemployed.

Then, examining the relationship between consumer personality (according to the Big Five model) and willingness to pay more provided initial evidence on this underexplored area of research. Highly conscientious consumers appear to be more willing to spend more for brands of low-priced products, probably due to their high organizational skills and reliability, which make them unlikely to change brands, even to the extent of paying a premium to remain loyal. They seem to be also inclined to pay a lower premium for mid- to high-priced products, presumably with an eye toward saving. Meanwhile, more open individuals might be willing to pay a higher premium for mid-high-priced products, possibly because of their characteristic trait of openness to experience.

Having discussed the various hypotheses, it seems important now to take a closer look at habit loyalty, as it stands out from other components of brand loyalty. Firstly, habit loyalty appears to be influenced by age and employment status but not by personality, unlike other subcomponents of brand loyalty. This suggests that habit loyalty may be driven by external factors—rather than psychological ones— such as financial availability, as indicated by age and employment status in this study. For instance, these two measures may have played a similar role within the questionnaire, providing an indirect indication of the participant’s financial availability or, in any case, the extent to which they can spend money on various purchases. Secondly, unlike other attitudinal components, habit loyalty does not appear to be a good predictor of loyalty behaviors, likely due to its lack of involvement. Furthermore, habit loyalty is the only brand loyalty subcomponent where higher values are associated with a lower intention to pay more for preferred low-priced brand products. In contrast, it does not affect the willingness to pay more for brands of mid- to high-priced products. This is consistent with the fact that habit loyalty scores were higher for the low-price range. The other brand loyalty components are likely more important for high-priced products, where higher consumer involvement has been shown to correlate with stronger brand loyalty (e.g., Ferreira & Coelho, 2015; Quester & Lin Lim, 2003). During the decision-making process related to purchasing a high-priced product, therefore, people may go through more extensive evaluations, not only because of a higher price but also because more expensive purchases are not made daily, while cheaper ones are made more frequently. This may explain why the premium amount (WTPMq) is influenced more by behavioral factors in the low-price range and by cognitive factors in the mid-high price range, where consumers engage in more cognitive evaluations, for example, regarding product quality. For these numerous reasons, this habit-based loyalty component deserves further investigation in future studies.

## 5. Conclusions

The current study confirmed the well-established causal relationship between brand loyalty and willingness to pay, as extensively documented in the literature. Further, it explored two relatively understudied topics, namely the relationship between brand loyalty and individual differences and between brand loyalty and product price range. While several associations emerged between personality traits and brand loyalty components, the major findings of this study pertain to the influence of product price range. The major findings in this study suggest that for higher-priced products, consumers are more likely to engage in both cognitive evaluations, such as assessing value for price, and affective evaluations, such as emotional attachment. In these cases, the intention to pay a premium is shaped by both factors, though the premium amount appears to be influenced more by cognitive considerations. In contrast, for lower-priced products, consumers may rely more on purchasing habits and economic availability, with loyalty and willingness to pay a premium driven more by familiarity and recommendations. However, further research is needed to confirm these patterns.

In addition to advancing the understanding of the psychological construct of brand loyalty and addressing pertinent research gaps, these findings could offer valuable insights for practical applications. Brands could utilize these insights by developing more targeted marketing strategies for distinct product price ranges. For mid- to high-priced products, companies could focus on emphasizing value propositions such as exceptional quality, unique design, and emotional resonance through storytelling and experiential marketing (Schmitt, 2012). For instance, luxury brands could continue to reinforce exclusivity and innovation in their messaging, appealing to cognitive evaluations of quality as well as the emotional significance of owning these products. For low-priced products, companies could implement strategies that leverage habitual purchasing patterns by offering promotions, loyalty rewards, or subscription models. For example, brands in the FMCG (Fast Moving Consumer Goods) sector could benefit from building frequently purchased products or using point-based loyalty systems to encourage repeat purchases. By tapping into habit-based loyalty, these strategies could strengthen brand-consumer relationships even in price-sensitive segments. Moreover, this study highlights the role of specific personality traits—Conscientiousness and Energy—in influencing loyalty. Brands could tailor their marketing efforts to target these traits, as these individual characteristics appear to predispose consumers to a specific form of consumer behavior, namely the formation of stronger bonds with brands, particularly in the mid- to high-price range. For instance, brands targeting conscientious consumers could highlight reliability, sustainability, and long-term value in their campaigns, as these elements align with the preferences of such individuals. Similarly, campaigns aimed at consumers high in Energy might focus on vibrant, dynamic content and interactive engagement through social media or gamified experiences, leveraging the energetic and novelty-seeking tendencies of this group.

In terms of policy implications, these findings suggest that policymakers could encourage transparency in marketing claims and pricing strategies, ensuring that consumers can make informed decisions, especially in the context of mid- to high-priced products where cognitive evaluations play a significant role. Additionally, public awareness campaigns could educate consumers on recognizing marketing techniques designed to appeal to personality traits, empowering them to make more autonomous purchasing decisions.

In conclusion, this study aimed to shed light on aspects of consumer behavior and the consumer-brand connection that have been less explored in the literature. The expectation is that future research will continue in this direction, delving deeper into psychological factors and mechanisms underlying consumer behavior and brand loyalty, and exploring how these insights can be applied to enhance both consumer satisfaction and brand profitability. By integrating findings into actionable strategies, both companies and policymakers have the potential to create value, foster loyalty, and promote sustainable consumer-brand relationships in a dynamic marketplace.

## 6. Limitations and Suggestions for Future Research

While this study provides valuable insights into consumer behavior, it is not without limitations. One significant limitation is the cross-sectional design, which restricts the ability to establish causal relationships between brand loyalty, personality traits, and product price range. Future studies could adopt a longitudinal approach to better capture the dynamic nature of these constructs over time (e.g., Menard, 2002; Rindfleisch et al., 2008). Additionally, reliance on self-reported data introduces the possibility of response biases, such as social desirability bias, which could influence the accuracy of the findings. Incorporating objective behavioral measures, such as purchase data or actual consumer observations through simulations, could provide more robust and reliable insights into these relationships (e.g., Chandon et al., 2005). A more balanced distribution of participants across socio-demographic categories would have facilitated more robust comparisons. Additionally, increasing the sample size would have allowed for a broader and more comprehensive selection of products across various price ranges, as well as a greater number of price categories for more detailed analysis. Future research should also aim to include income-related data, despite its sensitivity, as this would enable a deeper exploration of how financial availability influences brand loyalty, particularly habit loyalty, across different price segments. Future research could address these limitations by examining brands that offer products across distinct price segments, such as smartphones and Bluetooth earbuds, or brands with both budget and premium lines. This approach would enable a more comprehensive investigation into how product price range influences consumer brand loyalty, and whether loyalty is primarily driven by the most expensive, most frequently purchased, most advertised, or favorite product. Furthermore, due to the sensitive nature of income data, this study opted not to include an item related to participants’ income. However, including such a measure in future research would allow a more precise analysis of how financial availability influences brand loyalty, particularly in relation to habit loyalty and the price range of the products examined.

Another potential avenue for future research is the exploration of alternative personality models. While the present study utilized the widely applied Big Five model, future studies could incorporate the HEXACO model (Ashton et al., 2004), which includes the Honesty-Humility trait, offering the potential to reveal new insights into how personality traits interact with product price range and habit loyalty.

This study explored understudied topics, such as the influence of individual personality and product price range on brand loyalty, without employing direct measures of actual consumer behavior. For instance, a suggestion for future studies could be using simulations to further develop this line of research by providing a more robust indication of the actual behavior. In addition to simulations, longitudinal studies could provide valuable insights into how brand loyalty evolves over time and how it is influenced by changing consumer preferences, product innovations, and market trends.

Additionally, incorporating qualitative methods such as interviews and focus groups would have enriched the current study by offering deeper contextual understanding of the quantitative data collected through questionnaires, allowing for more comprehensive interpretation and insight.

Future research could examine consumers’ self-expression, as certain products and brands represent specific values (e.g., Voorn et al., 2021) or have become true status symbols (e.g., Ahad & Anshari, 2017), with particular attention to luxury brands (e.g., D. Choi et al., 2022). This line of inquiry could provide insights into how consumers’ evaluations differ across various product price ranges.

Moreover, habit-based loyalty, which plays a significant role in the lower-priced segment, deserves further investigation. The findings of this study suggest that this type of loyalty may be shaped by external factors, such as economic availability, which were not considered in this research. Further studies could investigate how situational factors, such as economic downturns or changes in disposable income, influence habit loyalty and purchasing behavior, particularly in price-sensitive segments.

## Figures and Tables

**Table 1:** Regression analysis: ABL → BBL (Source: Authors).

Product Price Range	IV→DV	β	*p*
Low	ABL→BBL	0.575	<0.001 ***
Mid-high	ABL→BBL	0.552	<0.001 ***
Overall	ABL→BBL	0.596	<0.001 ***

Abbreviations: ABL, attitudinal loyalty; BBL, behavioral loyalty; DV, dependent variable; IV, independent variable. *** *p* ≤ 0.001.

**Table 2:** Regression analysis: ABL subcomponents → BBL (Source: Authors).

Product Price Range	IV→DV	β	*p*
Low	aABL→BBL	0.446	<0.001 ***
	cABL→BBL	0.342	<0.001 ***
	hABL→BBL	−0.028	0.513
Mid-high	ABL→BBL	0.367	<0.001 ***
	cABL→BBL	0.426	<0.001 ***
	hABL→BBL	−0.024	0.574
Overall	ABL→BBL	0.412	<0.001 ***
	cABL→BBL	0.441	<0.001 ***
	hABL→BBL	−0.036	0.404

Abbreviations: aABL, affective loyalty; BBL, behavioral loyalty; cABL, cognitive loyalty; DV, dependent variable; hABL, habit loyalty; IV, independent variable. *** *p* ≤ 0.001.

**Table 3:** Regression analysis: BL → WTPMi (Source: Authors).

Product Price Range	IV→DV	*OR*	*p*
Low	BL→WTPMi	1.571	<0.001 ***
Mid-high	BL→WTPMi	1.830	<0.001 ***

Abbreviations: BL, brand loyalty; cABL, DV, dependent variable; IV, independent variable; WTPMi, willingness to pay more first item. *** *p* ≤ 0.001.

**Table 4:** Regression analysis: BL subcomponents → WTPMi (Source: Authors).

Product Price Range	IV→DV	*OR*	*p*
Low	aABL→WTPMi	1.637	<0.001 ***
	cABL→WTPMi	1.734	<0.001 ***
	hABL→WTPMi	0.697	0.005 **
	ABL→WTPMi	1.398	0.007 **
	BBL→WTPMi	1.815	<0.001 ***
Mid-high	aABL→WTPMi	1.652	<0.001 ***
	cABL→WTPMi	1.689	<0.001 ***
	hABL→WTPMi	1.081	0.549
	ABL→WTPMi	1.763	<0.001 ***
	BBL→WTPMi	1.579	<0.001 ***

Abbreviations: ABL, attitudinal loyalty; aABL, affective loyalty; BBL, behavioral loyalty; cABL, cognitive loyalty; DV, dependent variable; hABL, habit loyalty; IV, independent variable; WTPMi, willingness to pay more first item. ** *p* ≤ 0.01; *** *p* ≤ 0.001.

## Data Availability

Data is contained within Supplementary Materials.

## Note

1 * *p* ≤ 0.05; ** *p* ≤ 0.01; *** *p* ≤ 0.001.

## Supplementary Materials

The following supporting information can be downloaded at: https://www.mdpi.com/article/10.3390/bs15020189/s1.

## Appendix A. Measurement Items

Affective Loyalty (adapted from Dapena-Baron et al., 2020)

1. I feel passionate about this brand
2. If someone says something negative about this brand, I feel like they are attacking me personally
3. I feel good when I think about this brand
4. I feel a connection to other people who also use this brand
5. The fact that I use this brand says something about me
6. I love sharing stories about this brand with others who use it

Cognitive Loyalty (adapted from Dapena-Baron et al., 2020)

1. I expect this brand to deliver on its promise every time
2. An important reason I like this brand is its quality
3. If this brand does not deliver as expected, I will be very upset
4. I value this brand for its superior performance
5. One key reason to repurchase this brand is that it works as expected

Habit Loyalty (adapted from Dapena-Baron et al., 2020)

1. I buy and will repurchase this brand out of habit
2. I buy and will repurchase this brand just because I am used to it
3. I buy and will repurchase this brand just because it is familiar
4. I buy and will repurchase this brand as long as it is easy to find
5. I will keep buying this brand just because it is convenient

Behavioral Loyalty (adapted from Y. G. Choi et al., 2017)

1. I would recommend this brand to friends and relatives
2. I intend to keep buying this product from this brand
3. If I need this product, this brand would be my preferred choice
4. I will speak positively about this brand
5. I intend to encourage other people to buy this product from this brand

Willingness to Pay More (original items developed for this study)

1. Are you willing to pay more for the product of your preferred brand compared to other brands?
2. Indicate how much more you would be willing to pay for the product from your preferred brand compared to others

Personality (adapted from Barbaranelli et al., 2007)

Now rate how well each of these adjectives describes you on a scale from 1 (not at all) to 5 (completely):

1. Affectionate
2. Immaginative
3. Patient
4. Unselfish
5. Optimistic
6. Innovative
7. Responsible
8. Determined
9. Calm
10. Relaxed
11. Creative
12. Scrupulous
13. Energetic
14. Original
15. Enterprising
16. Dominant
17. Precise
18. Cordial
19. Conscientious
20. Resolute
21. Friendly
22. Diligent
23. Loyal
24. Modern
25. Serene
